# Single-Molecule Microfluidics for Membrane Protein Biophysics

**DOI:** 10.1007/s00232-026-00394-3

**Published:** 2026-09-08

**Authors:** Georg Krainer

**Affiliations:** 1 Biophysics, Institute of Molecular Biosciences (IMB), NAWI Graz, University of Graz, Humboldtstr. 50/III, Graz, 8010 Austria; 2 BioTechMed-Graz, Graz, 8010 Austria; 3 Field of Excellence BioHealth, University of Graz, Graz, 8010 Austria

## Abstract

**Figure:**
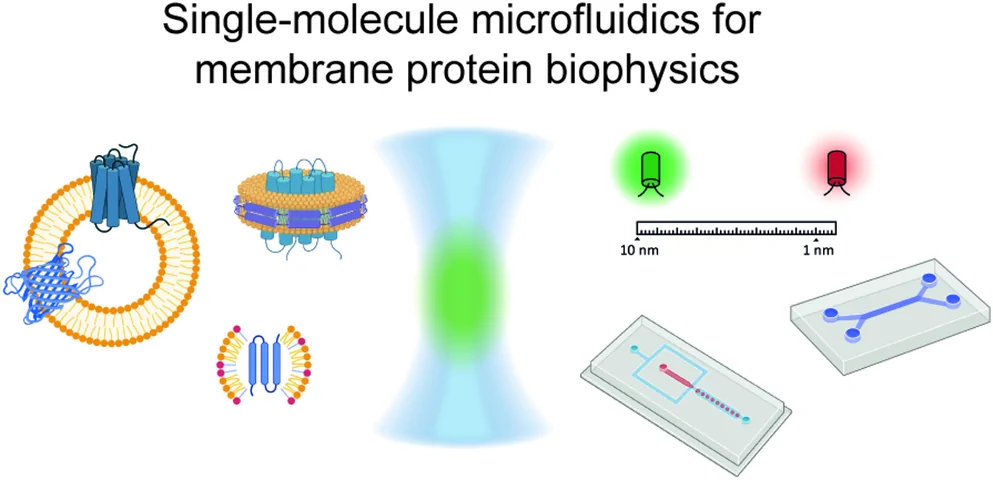

**Figure:**
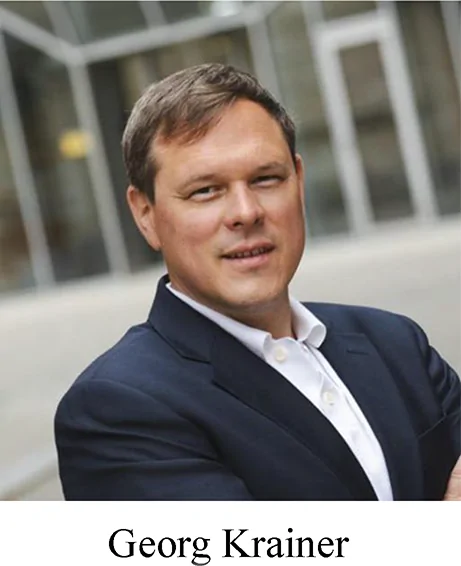

## Foreword by Sandro Keller

It is my great pleasure to introduce the following essay by Dr. Georg Krainer, featured in this issue’s ‘Up-and-Coming Scientist’ section of the *Journal of Membrane Biology*. Dr. Krainer is an Assistant Professor of Molecular Biophysics at the University of Graz, Austria, whose work brings together membrane-protein biochemistry, single-molecule fluorescence spectroscopy, and microfluidics.

Dr. Krainer studied Biochemistry at the Free University of Berlin, Germany, and completed his doctoral degree in Biophysics (*summa cum laude*) at the University of Kaiserslautern and the Technical University of Dresden, Germany. He then moved to the University of Cambridge, UK, for his postdoctoral research, where he was supported by Marie Skłodowska-Curie and Herchel Smith fellowships. In 2023, he joined the University of Graz as a research group leader and, following the award of an ERC Starting Grant, became Assistant Professor of Molecular Biophysics.

His research focuses on developing quantitative approaches to study protein dynamics, molecular interactions, and membrane-protein signaling under well-defined solution conditions. A particular strength of his work is the integration of methodological innovation with biologically important questions, especially in the context of membrane receptors.

In the following essay, Dr. Krainer discusses how single-molecule and microfluidic approaches can expand the experimental toolkit for membrane-protein biophysics. He highlights emerging opportunities to combine single-molecule Förster resonance energy transfer (smFRET), microfluidic perturbation, diffusional sizing, electrophoretic separation, and droplet-based assays to connect conformational dynamics, molecular interactions, and functional readouts at the level of individual molecules.

I am delighted to feature Dr. Georg Krainer in this Up-and-Coming Scientist contribution and look forward to the future impact of his work in membrane-protein biophysics.

## Introduction: Toward Dynamic Single-Molecule Microfluidics for Membrane Protein Biophysics

Membrane proteins are central to cellular physiology and pharmacology, controlling communication across membranes, molecular transport, and signal transduction, while also representing major therapeutic target classes (Stillwell 2016). Despite this importance, they remain challenging systems for quantitative biophysical analysis because they are difficult to produce, purify, and reconstitute, and because their function depends sensitively on the membrane environment, including lipid composition, curvature, and crowding (Cournia et al. 2015; Levental and Lyman 2022). Structural biology has transformed the field by providing atomic models of receptors, channels, and transporters in multiple states (Bajaj 2025), but structure alone cannot fully explain function. Receptor signaling, transporter cycling, and ion-channel gating depend on dynamic redistribution between conformations, transition rates, coupling to ligands or interaction partners, and stabilization by the membrane environment (Latorraca et al. 2016; Drew and Boudker 2016; Yan and Liu 2025). Mechanistic understanding therefore requires approaches that resolve how molecules move through conformational landscapes, how these motions are coupled to interactions, and how they respond to defined biochemical perturbations.

Single-molecule fluorescence methods are particularly powerful in this context because they avoid ensemble averaging. They can reveal subpopulations, rare states, dynamic transitions, and molecule-to-molecule variation. Among these methods, single-molecule Förster resonance energy transfer (smFRET) (Ha et al. 1996) has become one of the most informative tools for studying protein conformational dynamics (Lerner et al. 2018), because it converts nanometer-scale distance changes into fluorescence signals that can be monitored at the level of individual molecules. In membrane protein research, smFRET can report on ligand-dependent receptor activation, transporter domain movements, channel gating-associated rearrangements, or the formation of signaling complexes (Krainer et al. 2019; Agbadaola et al. 2025). Yet conformational dynamics are only one part of the problem. Membrane protein function is also shaped by molecular interactions, complex formation, oligomeric state, local membrane environment, and sample heterogeneity.

Microfluidics offers a complementary set of capabilities that can address many of these challenges (Streets and Huang 2014). By controlling fluids at the micro- and nanoscale, microfluidic devices enable precise manipulation of molecular transport, reaction timing, concentration gradients, mixing, confinement, and separation (Arter et al. 2020; Schneider et al. 2025). This is especially valuable for membrane protein studies, where samples are often scarce and where experiments require careful control over ligands, ions, nucleotides, detergents, lipids, or membrane mimetics. Microfluidic integration can reduce sample consumption, enable rapid or programmable perturbations, transform diffusion and electrophoretic mobility into quantitative biophysical readouts, and provide compartmentalized formats for screening and functional assays.

This essay discusses how combining single-molecule fluorescence with microfluidic control could expand the experimental toolkit for membrane protein biophysics. The focus is on approaches that link conformational dynamics, molecular interactions, and functional state under well-defined solution conditions. I first discuss smFRET as a reporter of membrane protein conformational dynamics and then consider how microfluidic perturbation can enable time-resolved measurements. I then highlight microfluidic single-molecule approaches for sizing, interaction analysis, and heterogeneity, followed by electrophoretic and droplet-based formats for separation, functional assays, and screening.

## Single-Molecule Fluorescence as a Central Reporter for Membrane Protein Conformational Dynamics

For many membrane proteins, function is best understood as redistribution across a conformational landscape. Ligands, ions, voltage, nucleotides, binding partners, and lipids do not simply turn proteins “on” or “off”; they shift the relative populations and lifetimes of molecular states. Single-molecule fluorescence techniques provide a direct way to observe such state distributions and transitions. Among them, smFRET offers a particularly powerful distance-sensitive readout (Krainer et al. 2019): two positions on a protein are labelled with donor and acceptor fluorophores, allowing distance changes in the 2–8 nm range to be monitored through changes in FRET efficiency (Lerner et al. 2018). Applied at the single-molecule level, smFRET reveals not only the average conformation, but also the heterogeneity and dynamics underlying the ensemble across biologically relevant timescales, from nanoseconds to seconds.

This capability is particularly important for G protein-coupled receptors, transporters, and ion channels. GPCRs are highly dynamic allosteric proteins that couple extracellular ligand binding to intracellular effector engagement (Hilger 2021). Their pharmacology is shaped by partial agonism, biased signaling, allosteric modulation, and ligand-specific stabilization of conformational states. smFRET is well suited to resolve these effects because it can distinguish inactive, intermediate, active, and effector-stabilized conformations and quantify how ligands reshape the conformational ensemble (Agbadaola et al. 2025). For transporters, smFRET can report on alternating-access transitions, substrate- and ion-dependent coupling, and the timing of domain rearrangements (Bartels et al. 2021). For ion channels, conformational FRET signals can complement electrophysiological readouts by showing how structural motions accompany gating (Martinac 2017).

A key advantage of smFRET is that it naturally captures heterogeneity. Membrane proteins often populate multiple states even under defined biochemical conditions. An ensemble measurement may suggest a single intermediate signal, whereas single-molecule trajectories may reveal distinct subpopulations or dynamic exchange between states. This is especially relevant for allosteric proteins, where ligand binding may not produce a uniform conformational response across all molecules. Instead, ligands can alter the probability of transitions, the lifetimes of states, or the coupling between domains. These effects are difficult to infer from ensemble averages but can be directly analyzed at the single-molecule level.

For membrane proteins, however, smFRET faces specific challenges (Krainer et al. 2019; Agbadaola et al. 2025). It usually requires site-specific labelling, often through mutations, removal of native reactive residues, or engineered tags, all of which can perturb folding, stability, ligand binding, conformational equilibria, or interactions with transducers and lipids. Additional complications include photobleaching, dye blinking, incomplete labelling, orientation effects, and uncertainty in converting FRET efficiencies into absolute distances.

The mode of interrogation also matters. Freely diffusing experiments are performed in solution and can capture fast dynamics, but they typically provide only short snapshots of individual molecules, often in the millisecond range (Hartmann et al. 2014). Surface immobilization can extend observation times but may introduce artefacts, including restricted conformational motion or non-specific interactions with the surface (Ruer et al. 2018). Moreover, many functionally relevant motions are small and difficult to resolve with available FRET pairs, highlighting the need for probes that can access shorter-range conformational changes.

In addition, membrane proteins must be studied in environments that preserve native-like behavior while remaining compatible with fluorescence detection. Detergent micelles and symmetric liposomes are convenient but may poorly recapitulate native membrane complexity, whereas nanodiscs and asymmetric lipid systems offer more native-like alternatives systems (Pabst and Keller 2024). Together, these challenges mean that smFRET experiments on membrane proteins require careful construct design, functional validation, suitable membrane mimetics, and orthogonal controls, and should be interpreted as probe- and environment-dependent molecular readouts.

## Microfluidic Perturbation and Time-resolved Single-Molecule Fluorescence

Many central questions in membrane protein biology are kinetic rather than equilibrium-based. How rapidly does a receptor respond to ligand binding? Which conformational intermediate appears first? How long does an active receptor remain competent for effector binding? How does substrate binding alter the transport cycle? How do ions, pH, or nucleotides alter the rates and pathways by which membrane proteins transition between conformational states? To answer such questions, single-molecule measurements must be combined with controlled perturbation.

Microfluidics provides an elegant route to such experiments. In laminar-flow devices, mixing can be controlled by diffusion across well-defined streams. In rapid-mixing geometries, reactions can be initiated on microsecond-to-millisecond timescales before molecules enter the observation region (Dingfelder et al. 2017). In droplet or segmented-flow formats (Yang et al. 2023), reactions can be initiated in isolated compartments, reducing surface adsorption and enabling many parallel reaction conditions. These strategies can transform single-molecule fluorescence approaches such as smFRET from equilibrium measurements into time-resolved assays that monitor molecular responses following a defined trigger.

For membrane proteins, possible triggers include ligands and modulators, ions, nucleotides, lipids, changes in pH, redox state, osmotic conditions, or interaction partners. In GPCR studies, for example, a microfluidic device could rapidly expose receptors to different ligands and monitor shifts in intracellular domain FRET signals. Subsequent addition of G proteins or other transducers could reveal how conformational states couple to effector engagement. For transporters, rapid substrate or ion jumps could be used to observe domain closure, alternating access, or coupling defects in disease-associated variants. For channels, ligand or ion jumps could be correlated with conformational rearrangements associated with activation, desensitization, or inactivation.

Microfluidic perturbation further enables experiments that go beyond simple relaxation kinetics after a single trigger. Many biological signaling processes are shaped by transient, repeated, or spatially varying inputs rather than by a single step change in conditions. By controlling not only the composition of a perturbation, but also its timing, duration, and sequence, microfluidics enables pulse–chase, ligand-exchange, recovery, or repeated-stimulation experiments. This makes it possible to ask whether membrane proteins return to their original conformational state, enter long-lived intermediates, or display path-dependent behavior. In this way, microfluidic single-molecule fluorescence can link molecular transition kinetics to the dynamic input patterns encountered in biological systems.

A particularly attractive future direction is repeated or programmable perturbation of the same molecular system (Kiselev et al. 2025). Instead of measuring a single ligand condition at a time, microfluidic devices could expose immobilized or trapped membrane proteins to sequences of ligands, effectors, or buffer conditions. This would allow direct comparison of state transitions within the same molecule or same field of view, reducing variability and enabling analysis of hysteresis, memory, desensitization, or adaptation. Such experiments would be especially valuable for receptors and transporters that do not simply return instantly to their initial state after stimulation.

However, microfluidic perturbation experiments also introduce specific challenges. The timing of a trigger must be calibrated carefully, because the observed fluorescence response depends not only on molecular kinetics but also on mixing times, flow profiles, diffusion, dead volumes, and the position of the observation region. For membrane proteins, additional complications include surface adsorption, slow ligand or lipid exchange within membrane mimetics, and possible destabilization by dilution, shear forces, or changes in detergent, lipid, or buffer composition. Time-resolved measurements also trade temporal resolution against photon statistics, observation time, and throughput, making rare intermediates difficult to quantify. Thus, such experiments require careful device characterization, kinetic controls, and comparison with orthogonal functional readouts.

## Single-Molecule Microfluidic Assays for Sizing, Interactions, and Heterogeneity

Membrane protein function is shaped not only by conformational dynamics, but also by molecular interactions. Reading out these interactions can provide insight into complex formation, binding stoichiometry, oligomeric state, and sample heterogeneity. Single-molecule fluorescence assays, including single-molecule counting, fluorescence correlation spectroscopy, and related approaches, can probe such processes in solution, but they also have limitations, including requirements for immobilization in some formats or challenges in fitting and interpreting complex correlation data. Recently developed single-molecule microfluidic assays offer an additional route to quantify molecular size, complex formation, and heterogeneity under controlled transport conditions.

**Fig. 1:**
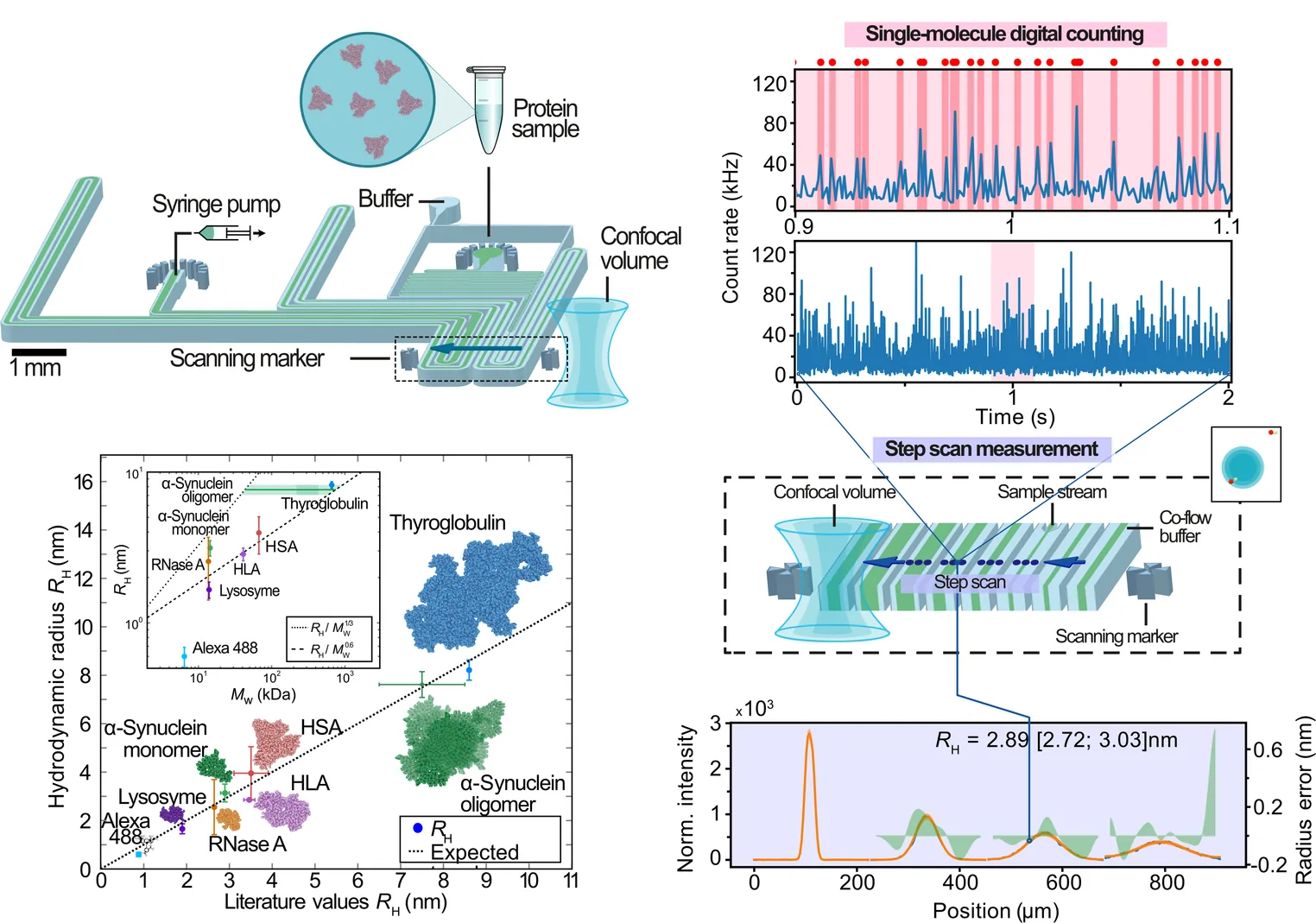
Single-molecule microfluidic diffusional sizing (smMDS). smMDS determines hydrodynamic size and molecular interactions from diffusion profiles measured by single-molecule fluorescence detection under microfluidic flow. Adapted from Krainer et al. 2024 under a CC BY license. © 2024 The Authors.

One such approach is single-molecule microfluidic diffusional sizing (smMDS) (Krainer et al. 2024), which leverages the relationship between molecular diffusion and hydrodynamic size (Fig. 1). In smMDS, fluorescent molecules are hydrodynamically focused into a narrow stream and allowed to diffuse laterally as they flow downstream through a microchannel. Smaller species diffuse more broadly, whereas larger species remain more confined. By scanning or imaging the distribution of individual fluorescent molecules across the channel, diffusion profiles can be reconstructed and converted into hydrodynamic radii. Because the readout is based on counting single molecules, smMDS can operate at very low concentrations, quantify low-abundance species, and resolve heterogeneous mixtures that would be difficult to analyze by ensemble methods.

For membrane protein biophysics, this type of assay offers several opportunities. One important application is the quantification of size and heterogeneity in membrane protein preparations. Membrane proteins in detergent, nanodiscs, liposomes, or polymer-stabilized particles often display distributions of particle size, aggregation state, or oligomeric state. Such heterogeneity can strongly influence downstream measurements, including smFRET, cryo-EM, activity assays, or pharmacological profiling. A sensitive solution-based sizing assay could therefore serve as a quality-control and mechanistic tool.

A related opportunity is the quantification of membrane protein interactions. Binding of an interaction partner, such as a ligand, nanobody, antibody, G protein, scaffold protein, or regulatory factor, can change the hydrodynamic radius of the fluorescently labelled species. Titration experiments can then be used to extract affinities or characterize binding stoichiometries, particularly when combined with appropriate models. For GPCRs, this could allow solution-based quantification of receptor-effector complex formation. For transporters, it could report on substrate-induced association with regulatory proteins or oligomerization-dependent activity. For channels, it could reveal assembly with auxiliary subunits or modulatory proteins.

Another advantage is the low sample requirement. Many membrane proteins are difficult to produce in large quantities, and highly purified, functional preparations may be available only at low concentrations. Methods that require minimal sample volume and operate at picomolar or femtomolar concentrations are therefore well matched to membrane protein research. This is especially important for newly discovered receptors, unstable transporters, human disease variants, or proteins extracted in native-like lipid environments.

Finally, such assays could be combined with conformational readouts. A molecule’s FRET efficiency reports on conformation, while its diffusion behavior reports on size or complex state. If both parameters can be measured in the same experiment, one could ask whether specific conformations correspond to particular assembly or binding states. For example, receptor molecules with high-FRET active conformations might be enriched in larger effector-bound complexes, whereas inactive conformations might remain monomeric or ligand-free. This type of multidimensional single-molecule analysis would move beyond simple population histograms toward a more mechanistic description of membrane protein function.

However, diffusion-based microfluidic assays also have limitations. Hydrodynamic radius is an effective transport parameter, not a direct measure of molecular mass or stoichiometry, and for membrane proteins it includes contributions from the surrounding detergent micelle, nanodisc, liposome, polymer, or lipid environment. Binding-induced size shifts may be small, particularly when the interaction partner is small relative to the membrane-mimetic scaffold, and overlapping diffusion profiles can make heterogeneous mixtures difficult to deconvolute. Thus, smMDS-type assays require appropriate physical models, controls for the membrane mimetic, and ideally orthogonal readouts such as fluorescence intensity, FRET, biochemical validation, or activity measurements.

## Electrophoretic Microfluidics and Mobility-based Interaction Analysis

Diffusion-based assays such as smMDS primarily report on size. Electrophoretic microfluidics adds a complementary molecular dimension: charge and electrophoretic mobility. In free-flow electrophoresis, molecules flow through a microfluidic chamber while an electric field is applied perpendicular to the direction of flow. Species with different electrophoretic mobilities are deflected to different positions, allowing separation and quantification in solution. When combined with fluorescence detection or single-molecule counting, this approach can distinguish free and bound species, resolve interaction states, and quantify low-abundance complexes.

For membrane protein studies, electrophoretic microfluidics could be useful in several ways. Many membrane protein interactions involve changes in charge, shape, or complex composition. Binding of charged ligands, nucleotides, peptides, nanobodies, antibodies, G proteins, arrestins, lipids, or scaffolding proteins can alter electrophoretic mobility. In a solution-based assay, such changes could be used to separate free and bound components without immobilization or washing. This is particularly attractive for weak or transient interactions, where immobilization can distort equilibria or where washing steps disrupt complexes.

**Fig. 2:**
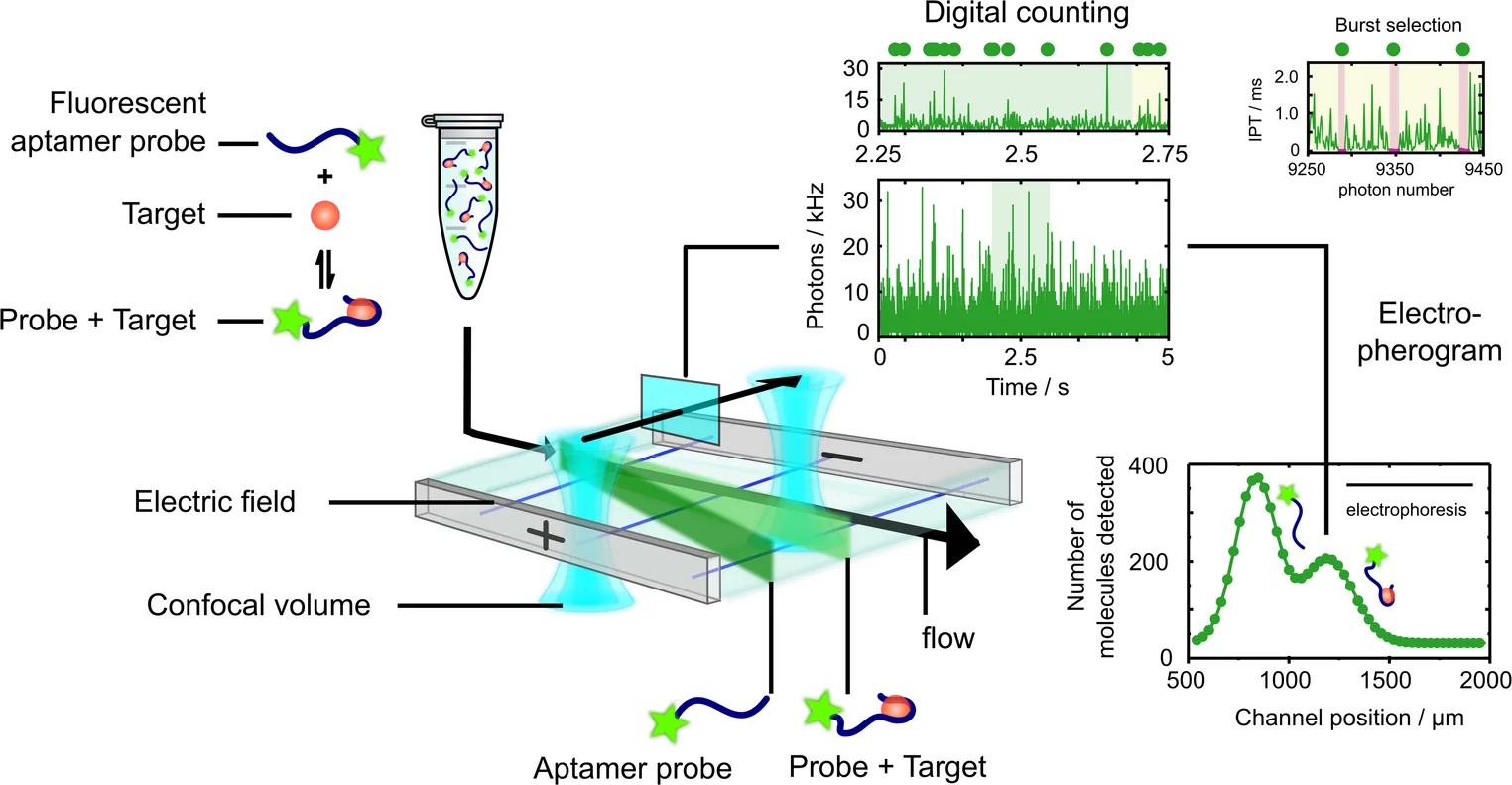
Digital Immunosensor Assay (DigitISA). DigitISA enables direct digital protein biomarker detection in solution by combining microchip electrophoretic separation with single-molecule fluorescence detection. Adapted from Krainer et al. 2023 under a CC BY license. © 2023 The Authors.

DigitISA (Krainer et al. 2023) is one example of such a mobility-based single-molecule assay, originally developed to quantify biomolecular interactions by electrophoretically separating free and target-bound fluorescent species before single-molecule counting (Fig. 2). DigitISA-like strategies therefore provide an especially interesting conceptual template for membrane protein studies. A fluorescent binder, aptamer, peptide, nanobody, or other recognition reagent can be mixed with a target, after which free and target-bound species are separated electrophoretically and counted at the single-molecule level. Applied to membrane proteins, analogous assays could quantify receptor–binder interactions, detect conformationally selective nanobodies, monitor ligand-induced effector recruitment, or screen for molecules that stabilize specific states.

Electrophoretic microfluidics may also help dissect heterogeneity in membrane protein preparations. Different particles can vary in lipid composition, oligomeric state, detergent or polymer content, bound cofactors, or associated proteins. These differences may produce mobility shifts even when size differences are small. Thus, electrophoretic separation could complement diffusional sizing, fluorescence intensity analysis, and FRET-based conformational classification.

There are, however, important challenges. Electrophoretic mobility depends on charge, size, shape, hydrodynamic drag, and buffer conditions, rather than reporting directly on a single molecular property. For membrane proteins, interpretation is further complicated by the amphiphilic environment: detergents, lipids, polymers, nanodiscs, or liposomes can contribute charge, heterogeneity, and electroosmotic behavior, and may dominate the mobility signal. Mobility shifts therefore cannot automatically be assigned to specific binding events, conformational states, or oligomeric assemblies without appropriate controls. Electric fields may also perturb fragile complexes or redistribute charged lipids and cofactors. Thus, electrophoretic microfluidics requires careful calibration, physical modeling, and orthogonal readouts such as diffusional sizing, fluorescence intensity, FRET, or biochemical binding assays.

## Droplet and Compartmentalized Microfluidics for Functional Assays and Screening

Beyond continuous-flow formats, droplet microfluidics offers another powerful route for combining single-molecule fluorescence with controlled biochemical experimentation (Moragues et al. 2023). In this format, reactions are compartmentalized into nanoliter- to femtolitre-scale droplets by dispersing an aqueous sample phase in an immiscible carrier phase, typically oil. This enables high-throughput screening, digital detection, and isolation of rare events. Although droplet technologies are often associated with enzyme assays (Gantz et al. 2023), nucleic-acid analysis (Ding et al. 2017), or diagnostics (Ma et al. 2026), they also offer opportunities for membrane protein research.

Femtodroplets are especially attractive for single-molecule studies because their volumes can approach the size of the confocal observation volume (Shim et al. 2013). This allows individual molecules or complexes to be interrogated efficiently as they pass through the detection region, while maintaining physically meaningful concentrations of binding partners. In this way, receptor–ligand, receptor–effector, or other membrane protein interactions could be preserved while retaining single-molecule resolution. When combined with fluorescence readouts such as smFRET, molecule counting, fluorescence intensity analysis, or burst analysis, droplet microfluidics could support high-throughput and low-volume analysis of membrane protein function and interactions. In combination with automation, different droplets could contain different ligands, lipid compositions, pH values, ions, nucleotides, or effector proteins, enabling sequential or parallel screening of conditions that alter conformational state distributions, complex formation, or membrane protein stability. Droplets can also function as isolated reaction chambers for time-resolved experiments (Yang et al. 2023): reagents can be mixed shortly before or during droplet generation, and droplets can then be observed after defined incubation times. This could allow ligand-induced conformational changes, effector recruitment, oligomerization, dissociation, or stabilization of rare states to be followed under controlled conditions.

In this way, compartmentalized microfluidics complements continuous-flow assays by enabling parallelized, concentration-controlled single-molecule experiments. At the same time, droplet approaches should not be overemphasized as a universal solution for membrane protein questions. Their small volumes and large interfacial area can introduce artefacts, including adsorption to the oil–water interface, incompatibility of membrane mimetics with the droplet formulation, and leakage or partitioning of hydrophobic ligands into the oil phase. These effects can alter effective concentrations, perturb binding equilibria, or destabilize fragile assemblies. Quantitative use of droplet assays therefore requires careful validation of droplet stability, reagent retention, membrane-mimetic compatibility, and comparison with bulk or continuous-flow measurements.

## Synthesis and Outlook

Membrane proteins rarely function through a single structural state or isolated molecular event. Their activity emerges from the coupling of conformational dynamics, ligand and effector binding, oligomeric state, membrane environment, and time-dependent perturbations. The methods discussed here address different parts of this problem: smFRET reports on conformational states and transitions, microfluidic perturbation introduces defined biochemical changes, diffusional sizing and electrophoretic separation report on molecular size, mobility, and complex formation, and droplet formats enable compartmentalized assays and screening. The next step is not simply to apply these methods in parallel, but to use them in combinations that connect these layers mechanistically.

This integration will be most powerful when guided by specific biological questions. For example, one may ask whether a ligand shifts a receptor into a conformation that is competent for effector binding, whether a transporter intermediate corresponds to a distinct oligomeric or lipid-associated state, or whether a membrane environment stabilizes a functional but low-population state. Such questions require more than a single readout. They require experiments in which perturbation, molecular state, interaction, and functional consequence can be related under defined conditions.

At the same time, the field will benefit from a realistic view of what each measurement reports. FRET efficiencies, diffusion profiles, electrophoretic mobilities, and droplet readouts are indirect observables that depend on probes, constructs, membrane mimetics, device geometry, transport processes, and data models. Their value therefore lies in complementing structural biology, biochemical assays, and functional measurements with molecularly resolved information that would otherwise be difficult to access.

In this sense, single-molecule microfluidics offers a route toward more dynamic and integrated membrane protein biophysics. Its most useful applications will likely be those in which methodological control and biochemical realism are balanced carefully: using small amounts of material, applying defined perturbations, resolving heterogeneity, and linking molecular behavior to mechanism. If developed in this direction, these approaches could help bridge the gap between static structural snapshots and ensemble functional assays, providing a clearer view of membrane proteins as dynamic, context-dependent molecular systems.

## Data Availability

No datasets were generated or analysed during the current study.
